# The status and correlates of depression and anxiety among breast-cancer survivors in Eastern China: a population-based, cross-sectional case–control study

**DOI:** 10.1186/1471-2458-14-326

**Published:** 2014-04-08

**Authors:** Feng Wang, Jiajia Liu, Liyuan Liu, Fei Wang, Zhongbing Ma, Dezong Gao, Qiang Zhang, Zhigang Yu

**Affiliations:** 1 Breast Disease Department, the Second Hospital of Shandong University, 247#, Beiyuan St, Tianqiao District, Jinan, Shandong 250033, P. R. China; 2 School of Nursing, Shandong University, No. 44 Wenhua Xi Road, Jinan, Shandong 250012, P. R. China; 3 School of Public Health, Shandong University, No. 44 Wenhua Xi Road, Jinan, Shandong 250012, P. R. China

**Keywords:** Breast cancer, Depression, Anxiety, Coping style, Social support, Predictor

## Abstract

**Background:**

Breast cancer presents specific challenges both physiologically and psychologically to women, and consequently affect the patients’ mental health. Psychosocial factors may play important roles in the symptoms and development of mental disorders among breast-cancer survivors. This study assesses the depression and anxiety status of breast-cancer survivors and further identifies the risk factors.

**Methods:**

A 1:1 matched, case–control study was conducted with a total sample of 222 individuals. Participants were selected from a national epidemiological survey. The Center for Epidemiological Studies—Depression Scale and State-Trait Anxiety Inventory were used to assess depression and anxiety. The Social Support Rating Scale and Perceived Social Support Scale were used as measures of social support and perceived social support, and the Rosenberg Self-Esteem Scale as a measure of self-esteem. Coping style was assessed using the Simplified Coping Style Questionnaire. The predictive effect of these psychosocial factors for depression and anxiety was investigated with hierarchical linear regressions.

**Results:**

Breast-cancer survivors experienced a high level of depressive and anxious symptoms. Multivariate analysis revealed that breast cancer functions as an independent but not a main risk factor of both depression and anxiety. Higher levels of depression and anxiety were positively associated with a higher level of passive coping style, and negatively with perceived social support, objective social support and an active coping style.

**Conclusions:**

The mental health of breast-cancer survivors should be promoted through the transformation of coping styles and improvement of social support.

## Background

Breast cancer is one of the most prevalent malignancies for women worldwide, especially in developed countries [1]. Compared with the high prevalence of breast cancer in Western countries, the incidence is relatively low in China. However, it has been reported that the incidence of breast cancer has sharply risen in recent years [2,3], which suggests much more attention should be given to breast cancer in China.

The advancement of medical technology has apparently increased the 5-year survival rate of breast cancer and made it possible for patients to live a prolonged life with long-term medication following surgery or chemotherapy. In addition to the prolonged lifetime, breast-cancer patients are also concerned with improved quality of life [4].

Breast cancer poses specific challenges both physiologically and psychologically to women. Specifically, intensive surgical and medical treatments destroy the integrity of the female body image, which consequently affects the patient’s mental health. An extensive literature review has shown that nearly a third to a half of female breast-cancer patients are likely to experience psychological distress [5], and up to one-third suffer from psychological morbidity, such as anxiety or depression [6-9]. Even after an effective treatment of the physical disease, such psychological distress may persist and accompany the patient for a long period, which has a dramatically negative impact on the patient’s quality of life [6,10]. Previous studies have shown that psychosocial factors play an important role in the symptoms and development of mental disorders. A longitudinal study of breast-cancer survivors has suggested that the course of the psychological symptom distress of a breast-cancer survivor is significantly affected by that of her supportive partner [11]. Another study found that a reduction in received support was related with a higher risk of mental disorders [12]. Deimling’s study revealed that passive coping styles such as denial can promote anxiety, depression and cancer-related worries [13]. In a recent research study conducted within a Chinese population, breast-cancer patients demonstrated greater psychopathology compared with healthy controls; however, other risk factors were not considered [14].

Taken together, previous studies on the quality of life and mental disorders of breast-cancer survivors usually consider only cases and seldom include healthy controls. Although psychosocial factors have often been studied among cancer survivors, most studies focus on a single factor. The objectives of the present study were twofold: (1) to compare women suffering from breast cancer with healthy control groups in terms of depression and anxiety status and (2) to further explore the psychosocial predictors for the development of psychological morbidity. Data used in the study were taken from a population-based, cross-sectional survey carried out in eastern China between July 15 and September 15, 2008.

## Methods

### Epidemiological survey

All data collected in the present study were taken from a cross-sectional survey carried out from July 15 to September 15, 2008, which was sponsored by the Ministry of Health of the People’s Republic of China. The survey was conducted in eastern China and covered 122,058 females in four provinces or metropolitan areas, namely Shandong, Jiangsu, Hebei and Tianjin. Random samples were selected through multistage stratified cluster sampling. The target population included 25 to 70-year-old females of the Han ethnic group with over two years of local residence at the time of the survey, and long-term migrant workers were excluded. Women who met the study requirements were selected for the wave survey. The wave I survey comprised face-to-face interviews of all subjects using a questionnaire, while only some subjects voluntarily completed psychological questionnaires in wave II. Details of the sampling procedure are explained at length elsewhere [15].

This study found 123 cases of breast-cancer patients who had been diagnosed within the past two years through the wave survey. Among these breast cancer cases, 51 (43.90%) were newly identified during the survey. All were screened and then voluntarily completed the wave questionnaire, which was designed to obtain the psychological status. In the end, 111 effective questionnaires were identified (cases) (a response rate of 90.24%) and matched with a control group of 111 healthy individuals. The study was approved by the ethics committees of the second hospital of Shandong University and those at the collaborating institutions in each region and informed consent was obtained from all study subjects.

### Identification of breast-cancer survivor cases and healthy controls

The inclusion criteria for breast-cancer survivor cases were 1) a diagnosis of breast cancer in the 2 years before the survey and 2) residence in the study area for at least 2 years.

The inclusion criteria for the healthy control group were 1) negative physical examination results, 2) negative ultrasound scans and/or mammographic screening results, 3) the same age as the cases (±2 years), 4) residence in the same area (neighbors and colleagues were the first choice) as cases for at least 2 years, and 5) no blood relationship with the breast cancer cases in case groups.

### Measures

#### Sociodemographic and medical factors

Sociodemographic and medical factors were measured employing a self-designed questionnaire. Sociodemographic status includes the age, body mass index (BMI), residence, education level (1: elementary or below; 2: junior high school; 3: senior high school; 4: college or above), marital status (1: never married; 2: ever married), and menopause status (yes or no). Basic diseases include diabetes mellitus and hypertension.

### Depression

Symptoms of depression were assessed using the Center for Epidemiological Studies—Depression Scale (CES-D). The scale consists of 20 items, of which four are reversely scored. Respondents rated their experience of each symptom in the last week on a 0–3 scale from “rarely or none of the time (on less than 1 day)” to “most or all of the time (on 5–7 days)”. The total score of the CES-D ranged from 0 to 60 with a cut-off score of 16. The CES-D has been used in numerous studies in China with satisfactory reliability and validity [16].

### Anxiety

Anxiety status was measured using the trait anxiety scale of the State-Trait Anxiety Inventory (STAI), which is composed of 20 items. Respondents were encouraged to report their general feelings about anxiety on a four-point scale: 1 (almost never), 2 (sometimes), 3 (often), and 4 (almost always). The total score reveals the anxiety level, ranging from 20 to 80. High scores indicate a high level of trait anxiety [17].

### Social support

The Social Support Rating Scale (SSRS) was adopted to evaluate social support. The SSRS was designed by Xiao [18] in 1990 on the basis of the Social Support Questionnaire [19] and the Interview Schedule for Social Interaction [20]. The scale consists of the three dimensions of objective support (three items), subjective support (four items) and the use of social support (three items).

### Perceived social support

The Perceived Social Support Scale (PSSS) [21] consists of 12 items, each of which is rated on a seven-point Likert scale. The PSSS emphasizes an individual’s self-understanding and self-perception of social support and measures the perceived level of the social support that individuals have received from family, friends and significant others separately through three subscale scores. The total score is accumulated for all the items and reflects the overall level of social support that an individual has perceived. A high score means a high level of perceived social support.

### Self-esteem

Self-esteem was evaluated by virtue of the Rosenberg Self-Esteem Scale (RSES). The RSES is the most widely used self-report instrument designed to measure global self-esteem, which should be understood as an individual’s overall evaluation of worthiness as a human being [22]. The RSES comprises 10 items scored on a four-point Likert scale that are accumulated to produce a single index of self-esteem. Higher scores represent higher levels of self-esteem [23].

### Coping style

The Simplified Coping Style Questionnaire (CSQ) consists of 20 items [24]. When the respondents are surveyed, a four-point Likert scale is used for each item, ranging from 0 to 3 (0: never do, 1: seldom do, 2: often do, 3: always do). This questionnaire is composed of two dimensions referred to as “active coping” and “passive coping”. Active coping covers the first 12 items, emphasizing the characteristics of the positive coping, such as “looking at the good side of things” and “finding several different solutions”. Covering the remaining eight items, passive coping emphasizes the characteristics of negative coping, such as “escaping troubles by drinking and smoking” and “dreaming that miracles could occur and the state quo could change”. A higher score for each dimension indicates frequent use of the coping style. The instrument has been found to have adequate reliability with Cronbach’s *α* = 0.89, and the internal consistency was found to have Cronbach’s *α* of 0.78.

### Data analysis

Epidata 3.1 (EpiData Association, Odense, Denmark) was used to establish the database. Data were analyzed using IBM SPSS Statistics 19.0 software (IBM Corporation, Somers, N.Y., USA). Continuous data were expressed as means with standard deviations, and the categorical data as frequencies. The case group and control group were compared in terms of demographic variables in *χ*^2^ tests and *t*-tests. Because there were no evidently significant differences, covariates were unnecessary. Categorical variables and continuous variables were compared through chi-squared analysis and a *t*-test respectively. Pearson correlation coefficients were used to explore the association of continuous variables. Results were considered to be of statistical significance if *P* < 0.05.

Hierarchical linear regressions were carried out, with the depression and anxiety score as the dependent variable, breast cancer entered in step 1 of the regression, and the remaining variables stepwise entered in step 2.

## Results

Sociodemographic and medical factors of the case group and control group are listed in Table 1. No differences were found between the two groups.

**Table 1:** Comparison of demographic characteristics of the case group and control group

	**Case group**	**Control group**	***χ/t***	***P***
**Age (SD)**	49.7 ± 9.6	49.2 ± 8.8	0.33(t)	0.74
**BMI**	25.1 ± 3.6	24.3 ± 3.6	1.54(t)	0.16
**Residence**			1.44	0.27
**Urban**	25(22.5%)	32(28.8%)		
**Rural**	86(77.5%)	79(71.2%)		
**Education level**			7.21	0.07
**Elementary or low**	49(44.2%)	51(46.0%)		
**Middle school**	29(26.1%)	34(30.6%)		
**High school**	18(16.2%)	22(19.8%)		
**College or high**	15(13.5%)	4(3.6%)		
Marital status			0.15	1.00
**Never**	3(2.7%)	4(3.6%)		
**Ever**	108(97.3%)	107(96.4%)		
**Menopause status**			2.86	0.11
**Yes**	58(52.3%)	46(41.4%)		
**No**	53(47.7%)	65(58.6%)		
Diabetes mellitus			3.69	0.12
**Yes**	6(5.4%)	1(0.9%)		
**No**	105(94.6%)	110(99.1%)		
Hypertension			0.00	1.00
**Yes**	14(12.6%)	14(12.6%)		
**No**	97(87.4%)	97(87.4%)		

Mean total and subscale scores of CES-D, STAI, SSRS, PSSS, RESE, and CSQ of the breast-cancer group and control group are presented in Table 2. Breast cancer survivors had much higher scores of CES-D, STAI, SSRS, subjective support, PSSS, and active coping of CSQ in bivariate analysis.

**Table 2:** Comparison of mean scores of CES-D, STAI, SSRS, PSSS, RESE, and CSQ for the case group and control group

	**Case group**	**Control group**	***t***	***p***
CES-D	16.64 ± 11.285	10.89 ± 9.271	4.074	0.000
**STAI**	42.34 ± 10.383	37.01 ± 9.212	4.035	0.000
**SSRS**	43.39 ± 5.548	45.68 ± 6.035	-3.075	0.003
**Objective support**	8.61 ± 2.301	9.03 ± 2.325	-1.333	0.186
**Subjective support**	27.78 ± 3.375	28.68 ± 3.338	-2.071	0.041
**PSSS**	63.64 ± 12.369	66.72 ± 10.261	-2.145	0.035
**RSES**	23.37 ± 2.679	22.72 ± 2.949	1.612	0.111
Active coping	19.69 ± 6.898	23.10 ± 7.158	-4.017	0.000
Passive coping	9.36 ± 4.270	9.73 ± 4.269	-.755	0.452

The Pearson correlations between total and subscale scores of SSRS, PSSS, RSES, CSQ, CES-D, STAI are shown in Table 3. All scores of these scales correlated closely with depression and anxiety, with the exception of self-esteem.

**Table 3:** Correlations between social support, perceived social support, self-esteem, and coping style and mental health

	**1**	**2**	**3**	**4**	**5**	**6**	**7**	**8**
**1. SSRS**	1							
**2. Objective support**	.684**	1						
**3. Subjective support**	.830**	.301**	1					
**4. utilization of social support**	.709**	.359**	.363**	1				
**5. PSSS**	.632**	.418**	.517**	.475**	1			
**6. RSES**	-.112	-.077	-.049	-.150*	-.012	1		
**7. Active coping**	.401**	.271**	.284**	.366**	.372**	-.205**	1	
**8. Passive coping**	-.039	-.019	.001	-.089	-.055	-.065	.178**	1
**9. CES-D**	-.425**	-.328**	-.287**	-.369**	-.472**	.031	-.371**	.276**
**10. STAI**	-.414**	-.328**	-.280**	-.351**	-.472**	.033	-.378**	.261**

Note: **p < 0.01, *p < 0.05.

Results of linear regression analysis are summarized in Table 4 (only including the variables entered in the models). In step 1, breast cancer was a significant contributor to both depression and anxiety with *β* values of 0.25 (*t* = 3.84, *p* < 0.001) and 0.27 (*t* = 4.20, *p* < 0.001), respectively, and accounted for 6.3% and 7.4% of the variance of depression and anxiety. In step 2, with the inclusion of other variables, the contribution to the models further increased by 33% and 30%. The contribution of breast cancer decreased (*β* = 0.16 for depression; *β* = 0.18 for anxiety) but was still of importance (*t* = 2.83 and 3.21, respectively; *p* < 0.005). As seen in Table 4, a higher level of passive coping was intimately associated with a higher level of depression and anxiety. Perceived social support, objective social support and an active coping style were found to be negatively associated with depression and anxiety.

**Table 4:** Multiple linear regression models for CES-D and STAI: standardized regression coefficients and standard errors

	**CES-D**		**STAI**	
	**β**	**SE**	**β**	**SE**
***Step 1***				
**Breast cancer**	.16**	1.17	.18**	1.14
***Step 2***				
**Perceived social support**	-.29**	.06	-.23**	.06
**Objective support**	-.12*	.27	-.14*	.27
**Passive coping**	.31**	.14	.30**	.14
**Active coping**	-.25**	.09	-.27**	.09

Note: **p < 0.01, *p < 0.05.

## Discussion

Previous studies have revealed that breast-cancer survivors are liable to be confronted with mental health problems, especially depressive and anxious symptoms, which are by no means abnormal responses to the intensive surgical and medical treatments, uncertainty, or loss of control. Although numerous researchers have focused on depression and anxiety morbidity in patients afflicted with breast cancer, to our knowledge, this is one of the few studies comparing breast-cancer patients with healthy controls to not only investigate psychological morbidity but also explore the psychosocial predictors for the development of depression and anxiety. Several previous studies have investigated breast-cancer survivors without healthy controls in group comparisons at different time points [9], or merely described physical and mental health statuses [25]. Most studies have explored influencing factors of depression and anxiety among breast-cancer survivors, paying attention to physiological predictors or single psychosocial factors [13,26]. The present study considered social support, perceived social support, self-esteem, and coping style as possible predictors of depression and anxiety.

The findings of this study confirm that breast-cancer survivors exhibit evidently higher depressive and anxious symptoms compared with healthy controls, which is consistent with previous research findings for the studied population [27]. The results show that, comparing with healthy controls, few breast-cancer survivors resort to active coping styles, which is similar to the findings of domestic research. Li et al. investigated breast-cancer patients in different age groups, and found that the elderly tended to employ active coping styles, while youth tended to adopt passive coping styles [28]. However, foreign researchers found that breast-cancer patients are more likely to face stress positively, instead of escaping from it passively [29]. This discrepancy can be interpreted as the consequence of different cultural backgrounds. Compared with healthy controls, individuals in the case group perceived less social support, which does not perfectly accord with the findings of previous studies [30,31] and is partly attributed to caregiver burnout. Long-term care may weaken the support provided by caregivers, who are unable to meet the constant demands of patients. Additionally, breast cancer restrains patients from frequent social activities, and support from the external world reduces as a result. At the same time, breast-cancer survivors need more care and support both materially and mentally than do healthy individuals, creating a large gap between expectations and reality.

Multivariate analysis demonstrated that breast cancer as an independent risk factor accounted for 6.3% and 7.4% of the variance of depression and anxiety. When other psychological factors were included, the predictive value increased considerably and cancer continued to be a significant contributor. This indicates that cancer is an independent predictor of depression and anxiety; however, the development of mental problems among breast-cancer survivors was in close relation with psychosocial factors such as social support, perceived social support and coping styles, which is partly consistent with the findings of previous studies [32]. These results suggest that breast cancer is not the main predictor of the development of depression and anxiety, although it can be considered an independent risk factor. Both coping styles and actual and perceived social support play important roles in the development of psychological problems. Therefore, as health promoters, we can advance the recovery of breast-cancer patients, especially psychological and social rehabilitation, through the transformation of coping styles and improvement of social support.

The present study suffers from a number of limitations. First of all, its cross-sectional design does not afford examination of participants’ changes over time. Without a longitudinal study it remains unclear whether and how breast cancer and mental disorder affects each other. Second, depression and anxiety status were assessed using self-report questionnaires without clinical interview, which may generate false positive or negative cases. Third, the correlates of psychological disorders could be affected by other factors not included in the present study. Another limitation is the absence of intervention, and further study is thus needed to generalize the findings. It is also unfortunate that we did not involve other meaning-related measures into our research, as the results could have shed further light on the relationship between psychological factors and breast cancer.

## Conclusions

The findings of this study showed that breast-cancer survivors exhibit evidently stronger depressive and anxious symptoms compared with healthy controls. They seldom adopt active coping styles, and they perceive there being less social support. Breast cancer is an independent predictor of depression and anxiety; however, coping styles and actual and perceived social support play important roles in the development of depressive and anxious symptoms. On the basis of these findings, we suggest that the mental health of breast-cancer survivors be promoted through the transformation of coping styles and improvement of social support.

## Competing interests

The authors declare they have no competing financial interests.

## Authors’ contributions

ZGY, JJL and Feng W conceived and designed the experiments. Feng W, ZBM, QZ, DZG and LYL performed the experiments. Fei W and LYL analyzed the data. Feng W, Fei W and JJL wrote the paper. All authors read and approved the final manuscript.

## Pre-publication history

The pre-publication history for this paper can be accessed here:

http://www.biomedcentral.com/1471-2458/14/326/prepub
